# Prediction of human prenatal exposure to bisphenol A and bisphenol A glucuronide from an ovine semi-physiological toxicokinetic model

**DOI:** 10.1038/s41598-017-15646-5

**Published:** 2017-11-10

**Authors:** Glenn Gauderat, Nicole Picard-Hagen, Pierre-Louis Toutain, Rémi Servien, Catherine Viguié, Sylvie Puel, Marlène Z. Lacroix, Tanguy Corbel, Alain Bousquet-Melou, Véronique Gayrard

**Affiliations:** 1 Toxalim, Université de Toulouse, INRA (Institut National de la Recherche Agronomique), INP (Institut National Polytechnique de Toulouse) –ENVT (Ecole Nationale Vétérinaire de Toulouse), Toulouse, France; 2 Agreenium’s International Research School (EIR-A), Paris, France

## Abstract

Bisphenol A (BPA) risk assessment is hampered by the difficulty of determining the extent of internal exposure in the human fetus and uncertainties regarding BPA toxicokinetics (TK) in the maternal-fetal unit. A feto-maternal TK model describing BPA and BPA glucuronide (BPAG) disposition in sheep was humanized, using human TK data obtained after d6-BPA administration on a cookie, to predict BPA and BPAG kinetics in the human mother-fetus unit. Validation of the model predictions included the assessed dose proportionality of BPA and BPAG disposition and the similarity between the simulated and measured time courses of BPA and BPAG in fetal rhesus monkeys after BPA maternal dosing. The model predicted fluctuations in fetal BPA concentrations associated with typical maternal exposure to BPA through the diet, with similar trough (0.011 ng/L vs 0.014 ng/L) and lower peak BPA concentrations (0.023 ng/L vs 0.14 ng/L) in fetal than in maternal plasma. BPAG concentrations in fetal plasma were predicted to increase over time to reach a steady value (29 ng/L) reflecting the cumulative BPA dose received by the fetus. Model-predicted BPAG concentrations in fetal plasma are consistent with reported levels in human cord blood that may be considered as relevant markers of the BPA dose entering blood throughout fetal life.

## Introduction

Bisphenol A (BPA) is an industrial chemical produced in high volumes and present in a wide variety of consumer products worldwide. As a result of its extensive and frequent use, over 90% of the human population in the US^1^, Canada^2^ and Europe^3^ is reported to be exposed to BPA. The risk assessment for BPA is controversial because the tolerable daily intake (4 µg/kg per day^4^) is higher than the doses shown to produce effects on laboratory animals, especially if dosing occurs during the perinatal period^5–7^. Resolution of the public health debate surrounding the risk for human health associated with fetal exposure to BPA largely depends on determining whether human fetal blood concentrations of unconjugated BPA are similar to or much lower than the concentrations measured in test species responding adversely to exposure.

Previous studies of BPA toxicokinetics (TK) in fetal sheep and rhesus monkey have shown that fetal Phase II metabolism leads to the production of conjugates, mainly BPA glucuronide (BPAG), which accumulate in the fetal circulation owing to their limited placental permeability^8–11^. Recently, the fetal sheep model enabled us to show that the slow elimination of BPAG through back conversion into BPA, results in a gradual re-entry of BPA into fetal blood^12^. This observation indicates that BPAG cannot be considered simply as an inactive irreversible terminal metabolite and underlines the need to characterize human fetal exposure not only in terms of static internal exposure to BPA and BPAG but also in dynamic terms to capture the back and forth exchanges between the BPA and BPAG pools.

The levels of BPA and BPAG concentrations in human fetal plasma are currently unknown. Biomonitoring studies conducted in neonates reported serum unconjugated BPA concentrations in the ng/mL range^13–17^. However, the reliability of such high BPA concentrations is questioned due to possible uncommon exposure conditions specifically related to exposure during delivery or to sample contamination post-exposure^18^. Given the inability of knowing univocally the internal exposure of the human fetus to BPA, this can at least be predicted by employing a TK modeling approach. According to the level and quality of information available, two types of TK models can be used: physiologically-based toxicokinetic models (PBTK) or compartmental models^19^. In PBTK models, the compartments represent actual tissue and organ spaces and their volumes are the physical volumes of those organs and tissues. PBTK models are therefore not only parameterized with chemical-specific parameters (chemical physico-chemical properties) but with (the) system-specific parameters that pertain to the anatomy and physiology of the organism^20^. The mechanistic basis of the PBTK parameters allows an extrapolation of TK estimates between species to be conducted by modifying the system-specific parameters such as blood flow, organ volume and composition. PBTK models have been developed for predicting BPA human exposure in adult humans and children^21–25^. Recently, Sarigiannis *et al*.^26^ developed a human mother-fetus PBTK model parameterized for BPA and BPAG which takes into account the changes in physiological and metabolic parameters that occur during gestation. Although these models have the potential to predict the plasma BPA concentration-time curve, they are complex and labor-intensive to build, and require a sound mechanistic basis that has not been well established for BPA and BPAG in the mother-fetal unit. Alternatively, a semi-physiologically-based BPA-BPAG compartmental model can be built, more simply and without a plurality of hypotheses, by physiological parameterization of a classical compartmental model^19^. Firstly, this makes it possible to specifically assess the relative contributions of metabolic and placental clearance processes to the fates of BPA and BPAG in the materno-fetal unit, and secondly to fit them to predict the fetal plasma BPA and BPAG concentration-time profiles. The value of using such semi-mechanistic models to predict undetectable BPA levels has been demonstrated in sheep^12^. Assuming that the fetal part of the model in sheep and humans is identical or at least similar, a humanized model can be obtained by scaling those models of BPA disposition developed in sheep by simply replacing the ewe maternal BPA and BPAG disposition parameters with those of humans, to predict the human BPA concentrations in fetal plasma associated with different exposure scenarios.

In the present study, a semi-physiologically-based TK model was specifically developed from data obtained in the feto-maternal sheep unit following maternal and fetal BPA or BPAG administrations. Sound evaluation of the fetal BPA and BPAG dispositions required the administration of high doses of BPA and BPAG and any extrapolations would be in the range of actually non-measurable plasma concentrations. We therefore first checked the validity of our modeling approach at low, more relevant, BPA exposure levels by demonstrating the dose- proportionality of fetal BPA and BPAG disposition. The adult part of the sheep model was then humanized by simply replacing the ovine maternal parameters with human parameters based on modeling raw data recently obtained in humans after exposure to BPA-d6 through the diet^27^. The generic predictive value of this feto-maternal humanized model was then assessed for its ability to predict the disposition of BPA and BPAG in pregnant rhesus monkey, a species for which the similarity of BPA and BPAG disposition with that of humans has been demonstrated^28^ and for which data exist for both fetal and maternal exposure after intravenous BPA maternal administration^9^. Finally, the final humanized model was used to predict BPA and BPAG plasma concentrations in the human feto-maternal unit under an exposure scheme corresponding to the latest EFSA epidemiological estimates of human external exposure to date.

## Results

The BPA and BPAG concentrations in all the control samples taken before dosing the test article were below the limits of quantification of the analytical method, thereby indicating that sample contamination had not occurred. All results are presented as mean ± SD.

**Figure 1:**
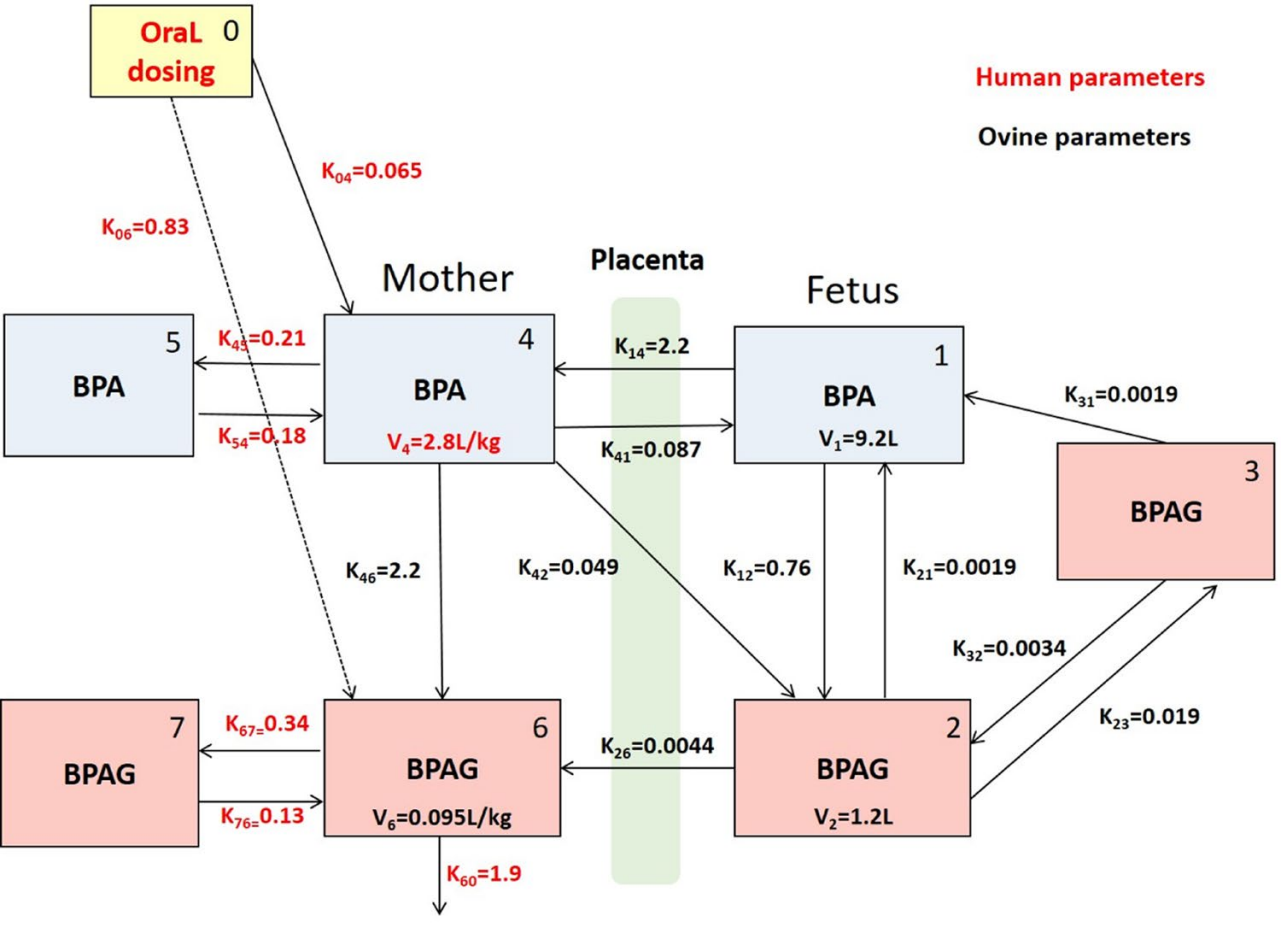
Schematic representation of the final feto-maternal humanized compartmental model. The fetal part of the model includes a central compartment for both BPA and BPAG designated 1 and 2 and a peripheral compartment (3) for BPAG. The maternal part of the model includes both a central and a peripheral compartment, respectively represented by 4 and 5 for BPA and by 6 and 7 for BPAG. Values above the arrows represent the first order rate constant of transfer between compartments (per hour). V1 and V4 represent the respective volumes of the fetal and maternal central compartments for BPA. V2 and V6 represent the respective volumes of the fetal and maternal central compartments for BPAG. The parameters of the maternal part of the model (V4, K45, K54, K67, K76 and K60) are those obtained in humans, as indicated in red; the other parameters of the maternal part of the model (V6, K46), the parameters of the fetal model (V1, V2, K12, K21, K23, K32, K31) and those linking the humanized maternal and ovine fetal models (K14, K41, K42, K26) are those obtained in sheep, as indicated in black.

A schematic representation of the final humanized feto-maternal model of BPA-BPAG disposition that was developed according to the different steps described below is given in Fig. 1.

### Dose proportionality of BPA and BPAG disposition in the fetus

Dose proportionality was investigated in 16 female and 14 male fetuses with a mean age of 118 ± 5 days and a mean body weight of 2.3 ± 0.7 kg. Details of the method of data analysis are given in Supplementary Information. As shown in Supplementary Fig. S1, the observed fetal plasma concentrations of BPA and BPAG could be fitted against the infusion rates of BPA and BPAG with a simple linear model. In addition, the intercept was shown to not be significantly different from 0, leading us to conclude dose proportionality for both BPA and BPAG disposition in the fetus (supplementary Table S1). Supplementary Fig. S2 shows the good agreement between observed and linear model-predicted concentrations. By taking the reciprocal of the slope calculated with the linear model describing BPA concentrations versus BPA infusion rates and BPAG concentrations against BPAG infusion rates, the computed estimates of fetal BPA and BPAG clearances^29^ were 22.5 L/h and 0.058 L/h, respectively.

### Contribution of the first pass conjugation of BPA to the fetal exposure to BPAG

As shown in Supplementary Fig. S3, the immediate detection of BPA-d6 in the fetal plasma following simultaneous fetal BPAG-d6 and maternal non-labeled BPA dosing indicates that part of the BPAG hydrolysis took place in the fetal central compartment. In addition, and importantly for the development of our model, the lower terminal slope of the time course of BPA-d6 concentrations in the fetal plasma, compared to that of BPAG-d6, indicates that BPAG-d6 hydrolysis occurs not only in the central BPAG compartment (rate constant designated K21) but also occurs in the peripheral BPAG compartment leading to a slow release of BPA-d6, that we then included in the model with a rate constant represented by K31 (Fig. 1). The ratios between the AUCs of the fetal BPAG and BPA plasma concentrations originating from maternal to fetal BPA transfer (364 and 407 for the two investigated fetal/maternal pairs in the present experiment) were higher than the corresponding ratios previously reported after direct BPA administration to fetuses (mean of 262 ± 119^12^). This result suggests that a fraction of the BPA transferred from mother to fetus had already been conjugated before reaching the systemic fetal circulation and this was taken into account for the model building (as a rate constant represented by K42, Fig. 1).

### Ovine feto-maternal modeling

The maternal sheep model, which was developed first, included both a central and a peripheral compartments, respectively designated 4 and 5 for BPA and 6 and 7 for BPAG, with a glucuronoconjugation constant between the BPA and BPAG central compartments represented by K46 (Fig. 1 and Supplementary Fig. S4). The central and peripheral BPA and BPAG compartments were interconnected with rate constants of transfer, respectively expressed as K45 and K54 for BPA and as K67 and K76 for BPAG. The BPAG elimination constant rate was designated K60. Supplementary Table S2 provides the BPA and BPAG population TK parameter estimates. The volume V4 of the maternal central compartment of BPA (0.815 L/kg) and the BPA plasma clearance, i.e. V4*K46 (1.75 L/(kg.h)) were both much higher than those of BPAG (V6 = 0.0945 L/kg and V4*K60 = 0.30 L/(kg.h)).

**Figure 2:**
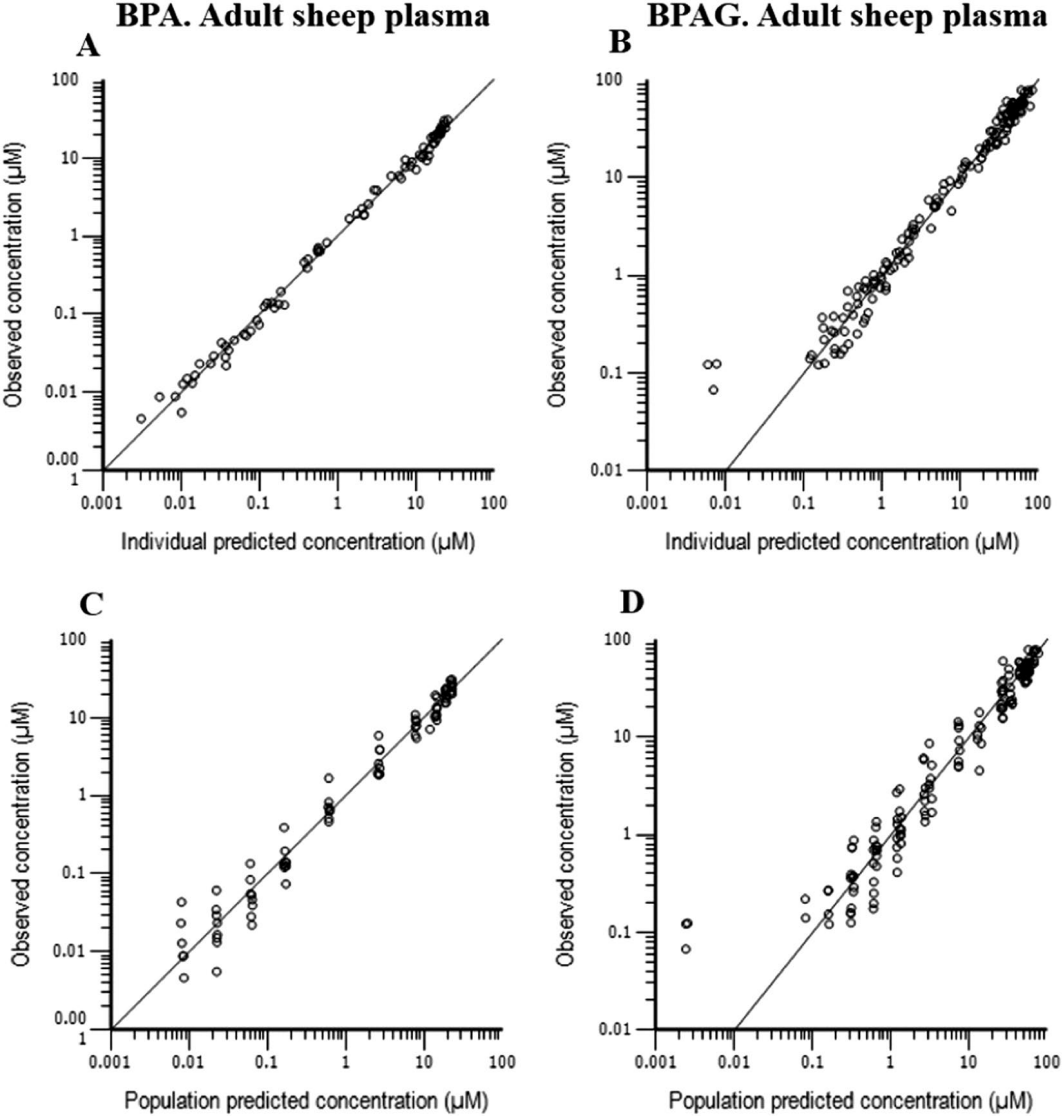
Diagnostic plots for the adult sheep (maternal) disposition model obtained after simultaneous fitting of BPA and BPAG plasma concentrations following BPA and BPAG IV administrations in 8 non-pregnant ewes. Individual predicted *vs*. observed BPA (**A**) and BPAG (**B**) plasma concentrations (µM); Population predicted *vs*. observed BPA (**C**) and BPAG (**D**) plasma concentrations (µM). For population predicted *vs*. observed concentrations, data were evenly distributed over the line of identity indicating that the selected structural model was appropriate. Individual predictions were obtained by including a random component in the “post-hoc” or empirical Bayesian estimate of random effect. For these individual predicted *vs*. observed data plots, the data were also evenly distributed around the line of identity suggesting the absence of any major bias in the random component of the model.

The between- subject variability (BSV) was modeled with an exponential model for all the structural parameters except the BPAG distribution parameters (K67 and K76) that could not be robustly estimated. The corresponding coefficients of variation (CV) ranged between 10 and 33% (Supplementary Table S2) indicating a rather good homogeneity of the investigated ewes. The goodness-of-fit plots (Fig. 2) show that the data were appropriately described by the model, thereby confirming that BPA elimination in ewe occurred almost exclusively through glucuronidation.

As shown in Fig. 1 and Supplementary Fig. S5, the fetal model grafted to the maternal sheep model included three compartments with a single central compartment for BPA (noted 1) and both a central and a peripheral compartment respectively represented by 2 and 3 for BPAG. The two central fetal compartments were interconnected with rate constants of transfer to describe glucuronoconjugation (from BPA to BPAG compartments represented by K12) and hydrolysis (from BPAG to BPA compartments represented by K21). In addition, a fetal BPAG hydrolysis constant was introduced between the peripheral BPAG compartment and the central BPA compartment (K31) to describe BPAG hydrolysis into BPA directly from the fetal tissular compartment, as shown by the results obtained after fetal BPAG-d6 administration (see above). The maternal and fetal BPA central compartments were directly linked in both directions (K14 and K41), whereas the maternal and fetal central compartments of BPAG were only linked in the fetal-to-maternal direction (rate constant represented by K26) since no BPAG was detected in fetal plasma after BPAG administration to the pregnant ewe. An additional rate constant between the maternal central compartment of BPA and the fetal central compartment of BPAG (K42) was added to account for the first-pass conjugation effect occurring between the maternal and fetal circulations.

**Figure 3:**
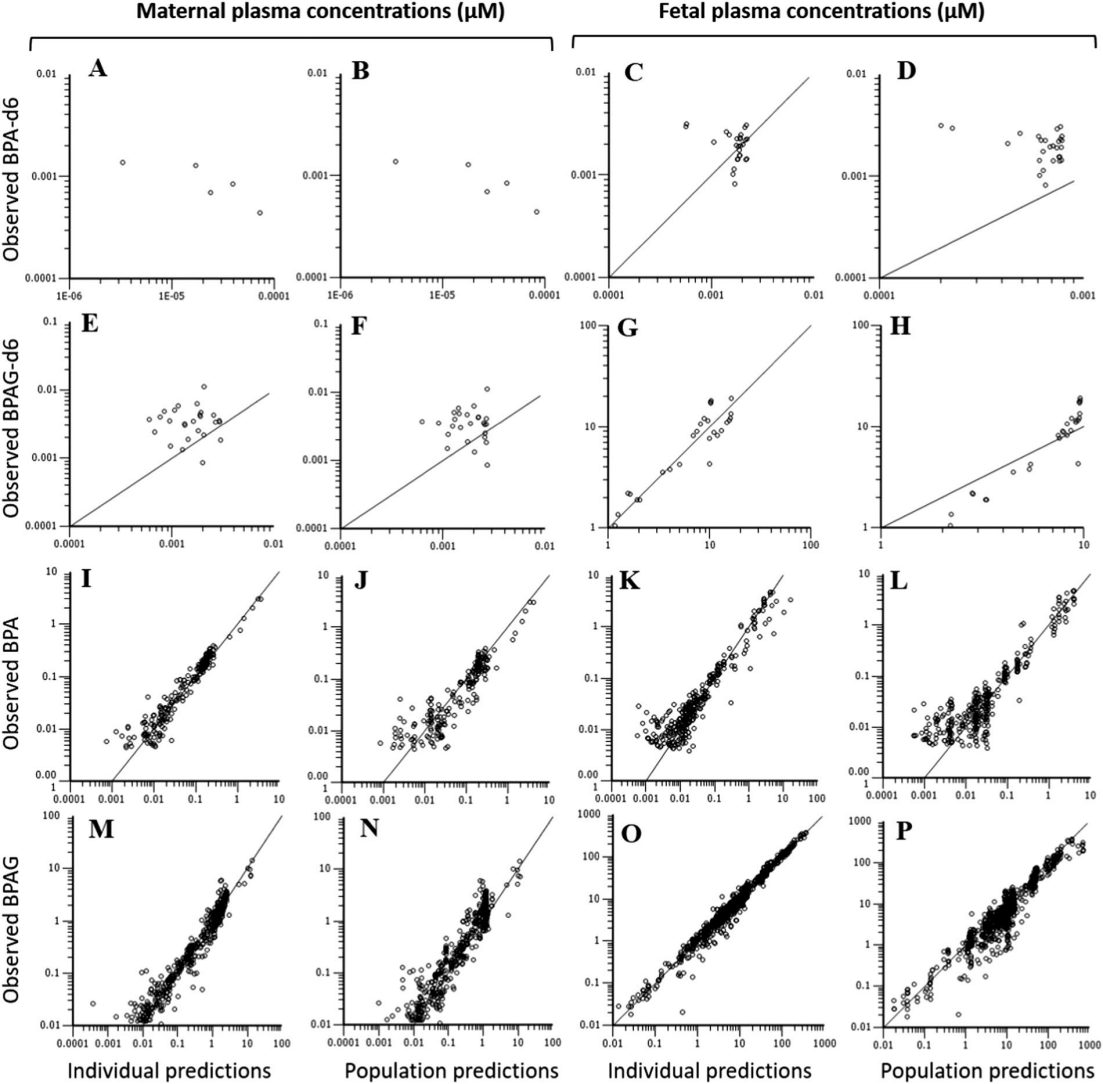
Diagnostic plots for the feto-maternal TK model obtained after simultaneous fitting of BPA, BPAG, BPA-d6 and BPAG-d6 concentrations (µM) in maternal and fetal plasma after various IV administrations (BPA and BPAG fetal and maternal bolus and infusions, BPAG-d6 fetal bolus). Analytes are grouped by line with individual and corresponding population predictions presented side by side (left and right panels respectively). (**A,E,I** and **M**) Individual predicted *vs*. observed respective BPA-d6, BPAG-d6, BPA and BPAG concentrations in maternal plasma; (**B,F,J** and **N**) Population predicted *vs*. observed respective BPA-d6, BPAG-d6, BPA and BPAG concentrations in maternal plasma; (**C,G,K** and **O**) Individual predicted *vs*. observed respective BPA-d6, BPAG-d6, BPA and BPAG concentrations in fetal plasma; (**D,H,L** and **P**) Population predicted *vs*. observed respective BPA-d6, BPAG-d6, BPA and BPAG concentrations in fetal plasma (see Fig. 2 for meanings of population vs. individual predictions).

The goodness-of-fit plots for fetal disposition of BPA and BPAG (Fig. 3) show that, overall, the feto-maternal model appropriately described the data. The only departure from the model was apparent for the BPA-d6 maternal plasma concentrations soon after BPAG-d6 fetal dosing. However, those outlier concentrations could originate from residual BPA-d6 contamination in the BPAG-d6 dose (estimated to be less than 1% of the fetal BPAG-d6 dose).

The computed feto-maternal population TK parameters (Supplementary Table S3) showed that the estimated maternal-to-fetal BPA transfer represented 6% of the total BPA maternal clearance, meaning that the fetus, in relation to its body weight, received a dose of BPA equivalent to the maternal dose. The fetal BPA conjugation clearance (i.e. K12*V1 = 7.0 L/h) represented 26% of the total BPA fetal clearance, the remaining BPA being eliminated through the fetal-to-maternal BPA transfer. Thirty-six % of the BPA transferred from mother to fetus was conjugated before reaching the fetal circulation, meaning that 69% of the total fetal BPAG exposure when BPA was administered to the mother, was due to this first-pass effect. BPAG hydrolysis from the central and peripheral fetal compartments represented by far the main route of definitive BPAG elimination from the fetus (83%), the estimated direct BPAG fetal-to-maternal transfer (K26) representing only 17% of the total BPAG outputs from the central fetal BPAG compartment.

### Human feto-maternal modeling

The compartmental representations of the adult part of the human BPA and BPAG models are shown in Fig. 1 and Supplementary Fig. S3. The model structure used to fit the human data consisted of the adult part of the ovine model to which was added an absorption sub-model. The oral BPA dose is administered in the absorption compartment (designated 0) and linked to the BPA and the BPAG central compartments with rate constants of transfer to describe respectively BPA systemic bioavailability (K04) and BPA first-pass conjugation (K06).

**Figure 4:**
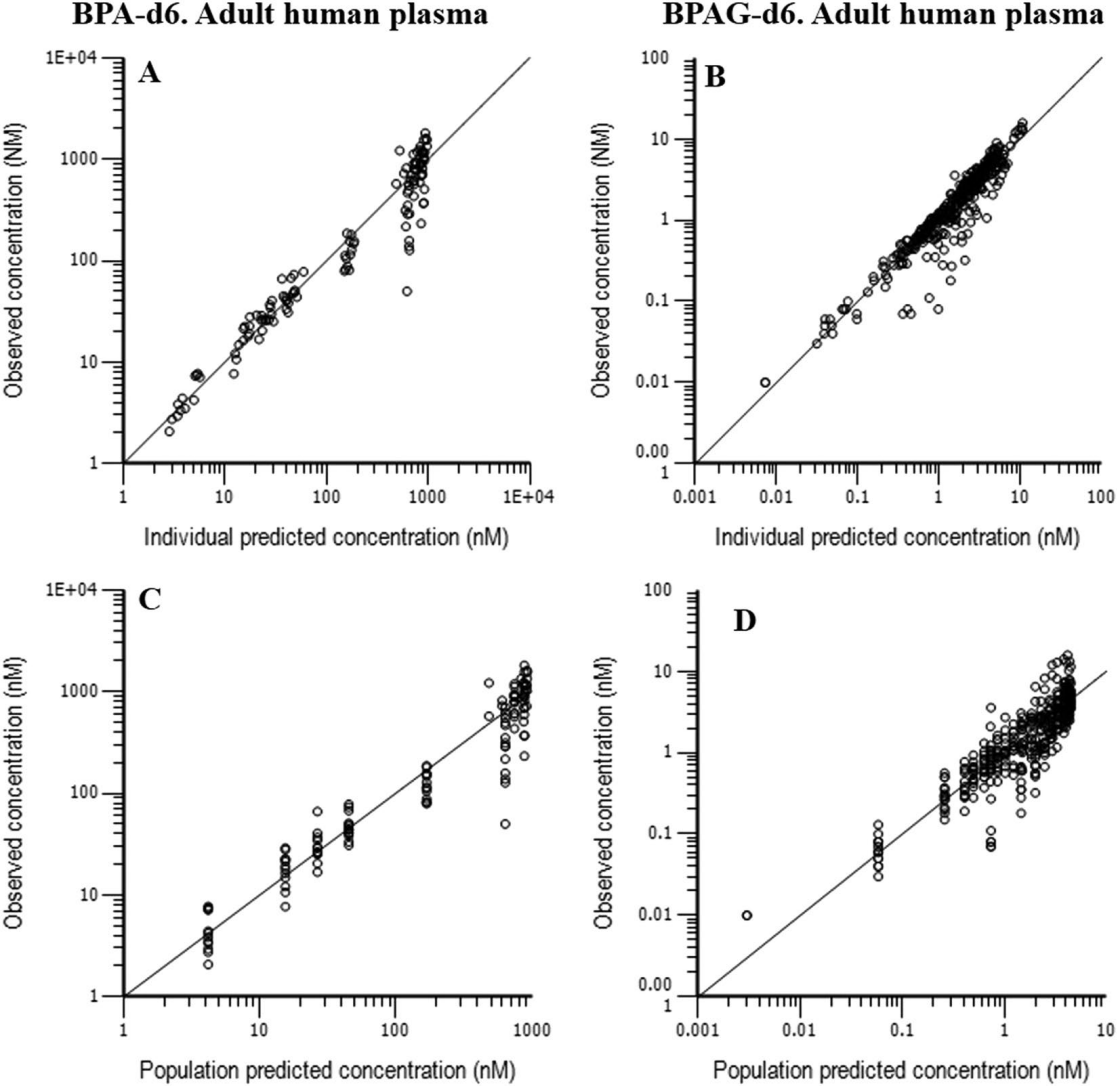
Diagnostic plots for the adult human disposition model obtained after simultaneous fitting of BPA-d6 and BPAG-d6 plasma concentrations (µM) following a BPA-d6 oral administration (Analysed raw data were obtained from Thayer *et al*.^27^). Individual predicted *vs*. observed BPA-d6 (**A**) and BPAG-d6 (**B**) plasma concentrations; Population predicted *vs*. observed BPA-d6 (**C**) and BPAG-d6 (**D**) plasma concentrations (see Fig. 2 for meanings of population and individual predicted concentrations).

The goodness of fit plots (Fig. 4) indicate that the structural model developed in adult sheep adequately modeled the raw data for BPA-d6 and total BPA-d6 (considered as BPAG-d6) plasma concentrations obtained after oral BPA-d6 administration in humans^27^.

The between-subject variability (BSV) was modeled using an exponential model for all the structural parameters except the conjugation rate (K46) and the BPAG central volume (V6, Supplementary Table S4). The estimated adult human and ovine parameters were of similar orders of magnitude (Supplementary Tables S2 and S4). The volume of the central compartment of BPA was higher in humans than in sheep (2.8 L/kg vs 0.815 L/kg) while the BPAG clearance was lower (0.18 vs 0.30 L/(kg.h)). By using a human BPA clearance of 1.536 L/(kg.h) estimated by allometric scaling^30^, analysis of the human raw data obtained from Thayer *et al*.^27^ indicated an absolute oral bioavailability of BPA of 7.3% and that 92.7% of the BPA-d6 dose administered by dietary route was directly glucuronoconjugated by a first-pass effect before reaching the systemic circulation.

The final humanized model used to predict human prenatal exposure to BPA and BPAG included the adult human model to which had been grafted the ovine maternal-to-fetal and the full ovine fetal models (Fig. 1).

### Evaluation of the humanized feto-maternal model

**Figure 5:**
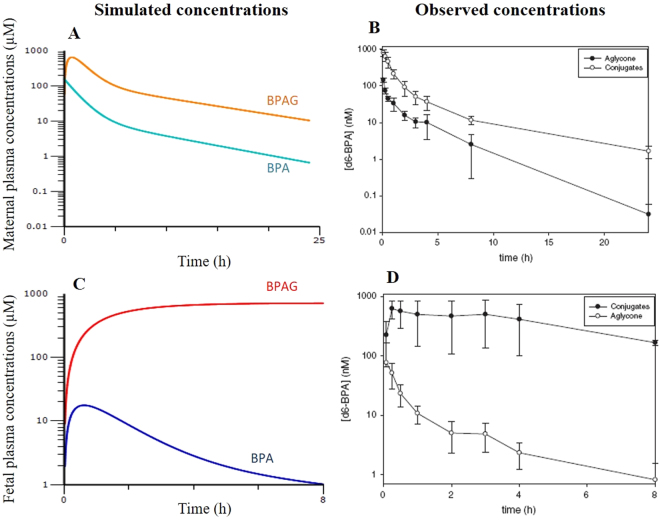
Semilogarithmic plots of model-simulated BPA and BPAG concentrations in maternal (**A**) and fetal plasma (**C**) using the final humanized model developed in the present study and observed concentrations of aglycone BPA and BPA conjugates in maternal (**B**) and fetal plasma (**D**) of rhesus monkeys after a single maternal BPA-d6 IV dose of 100 μg/kg^9^.

Figure 5 shows the model-predicted and observed aglycone and conjugated BPA-d6 concentrations in the fetal and maternal plasma of rhesus monkey after maternal 100 µg/kg BPA-d6 IV dosing^9^. The predicted time course of fetal and maternal BPA and BPAG concentrations fitted well with the published decay of TK data^9^, apart from the first 30 min following maternal dosing when the BPA-d6 concentrations decreased in fetal plasma while the simulated concentrations increased to a peak value 30 min after dosing.

### Human BPA and BPAG exposure predictions for the EFSA average external daily exposure in human

**Figure 6:**
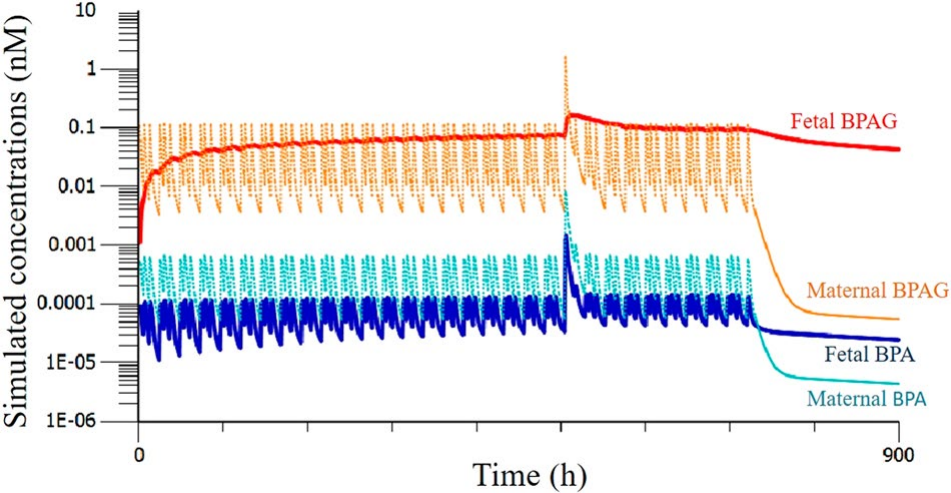
Model-predicted BPA (cold colors) and BPAG (warm colors) concentrations (nM) in fetal (thick solid lines) and maternal plasma (thin dotted lines) under a maternal exposure scenario corresponding to the EFSA’s average daily exposure estimates^4^ (11.05 nmol/d for 70 kg body weight) split into 3 doses a day. A single high dose (55.85 nmol) was included after 3 weeks of exposure.

Figure 6 shows the simulated BPA and BPAG concentrations in human maternal and fetal plasma during 4 weeks of daily BPA oral dosing at a level equivalent to the mean daily exposure estimated by EFSA^4^ which also included a high single dose corresponding to the estimated EFSA 95 th percentile of human exposure.

The model predicted a substantial accumulation of BPAG (about 20 fold) in fetal plasma, leading to relatively steady BPAG concentrations that reached 29 ng/L after 3 weeks of BPA exposure, while the corresponding concentrations in maternal plasma fluctuated between 1.6 and 40 ng/L. The peak BPA concentrations associated with BPA intake did not increase over time, exhibiting 6 times higher values in maternal plasma (0.14 ng/L) than in fetal plasma (0.023 ng/L), while BPAG accumulation and subsequent hydrolysis led to an increase of BPA trough concentrations in the plasma that attained similar values in both the maternal and fetal plasma (0.014 vs 0.011 ng/L).

Delivery of a single high maternal BPA dose (x15) had long term effects on fetal BPAG and BPA basal exposure but only induced a transient increase in the maternal concentrations. After this dose, the BPAG fetal plasma concentrations peaked at 64 ng/L then slowly decreased to reach 44 ng/L one week later. A week after the final administration, the sustained BPA and BPAG concentrations were respectively 5 and 780 times higher in the fetal plasma (4.3 pg/L and 14 ng/L) than in the maternal plasma (0.8 pg/L and 0.018 ng/L).

## Discussion

The present article, using a humanized semi-physiological compartmental model of BPA and BPAG disposition predicted, from reported external BPA exposure in human, that human fetal exposure to BPA would be within a range similar to the maternal concentrations but also suggests that BPAG would be strongly accumulated in human fetuses. Our approach was based on compartmental modeling of the feto-maternal TK of BPA and BPAG in sheep, because rich data sets are available for these two analytes in this species (both in the fetal and maternal compartments) which circumvents the need to postulate the numerous assumptions required to build a more generic PBTK model. The ovine model was then used to predict human fetal exposure by substituting maternal ovine parameters by human ones obtained by analyzing oral raw data recently published for humans^27^. The reliability of the ovine feto-maternal model to predict fetal plasma concentrations associated with low BPA exposure levels was ensured by assessing the dose-proportionality of fetal BPA and BPAG disposition for a wide range of doses. The pregnant sheep was selected as a relevant generic model for the extrapolation of BPA fetal kinetics to the human fetus because it is an acknowledged model for studying nutrient exchange^31^ and characterizing drug disposition during the prenatal period^32,33^. Furthermore, BPA glucuronidation in humans and sheep is very similar, and near total in adults^8,12,34^. The relevance of the sheep model is also comforted by the similar mechanisms of BPA placental clearances^35^.

By developing the ovine feto-maternal model, we were able to quantify the relative contributions of the different pathways controlling fetal exposure to BPA and BPAG. We showed that fetal exposure to BPA and BPAG is mainly dependent on the level of the bioavailable maternal BPA dose, the maternal to fetal transfer of BPA representing 6% of the BPA maternal dose, *i.e*. a value of the same order of magnitude as that estimated from the *ex vivo* model of perfused human placenta^35^. We estimated that the human oral bioavailable BPA dose represented 7.3% of the orally ingested BPA. This is similar to the estimated oral bioavailability in rhesus monkeys (7.3%) after BPA administration in a piece of fruit^11^, but much higher than the 0.2% measured after BPA administration by gavage in the same animal model^36^. In accordance with the observations in other species, including dogs^37^ and sheep^38^, but at variance with the values routinely quoted in man, this result strongly suggests that buccal absorption, that avoids an hepatic first pass effect, is an important entry route in humans by which BPA in food can gain direct access to the systemic circulation.

The model supports the major influence of direct clearance of BPA from the fetus to the mother on the BPA plasma concentrations in fetal blood. Indeed, placental BPA clearance represents about 74% of the fetal BPA clearance, as previously shown in fetal sheep^8,12^. The remaining part of the elimination of BPA from the fetal compartment (26%) is accounted for by fetal metabolism of BPA into BPAG. Therefore, BPAG fetal exposure results from fetal BPA metabolism and indirectly from the BPA dose transferred from the mother to the fetus since the maternal to fetal clearance of BPAG in sheep was shown to be negligible, in agreement with the extremely limited placental permeability observed in humans^8^. Our results indicate that significant conjugation activity occurs in the sheep fetus. Indeed, BPA conjugation clearance in fetal sheep (7 L/h or 2.8 L/h.kg) is higher than the adult BPA clearance (1.75 L/h.kg) although the intrinsic hepatic glucuronidation clearance in late fetal sheep is nearly 2 times less than in adults^39^. The BPA fetal clearance value was close to the estimated fetal hepatic blood flow of 5.1 L/h, obtained by combining the portal vein and hepatic artery flows^40^ implying that fetal BPA conjugation is blood flow-limited. These observations suggest that although fetal expression of the UGT involved in BPA glucuronoconjugation is less in the human fetus than in adults^41^, BPA clearance in late pregnancy fetus is limited by the hepatic fetal blood flow. The high BPA clearance by the fetal liver is also responsible for the significant first-pass conjugation of BPA transferred from the mother to the fetus (modeled as a rate constant (designated K42) that increases the fetal exposure to BPAG. Hence, fetal exposure to BPAG is 69% higher after maternal BPA dosing than after direct IV administration of the same molar BPA dose to the fetus. This placenta-hepatic first-pass conjugation may be as important in humans as in sheep since the median fraction of umbilical blood flow that reaches the fetal liver before joining the systemic fetal circulation has been estimated at 79%^42^.

Fetal clearance of BPAG across the ovine placenta (rate constant designated K26) was low, accounting for 17% of the BPAG fetal clearance. BPAG was shown to be mainly eliminated through the fetal to maternal transfer of BPA resulting from BPAG hydrolysis. The mechanisms of human fetal BPAG clearance are currently unknown and no deconjugation data is available for humans or non human primates. Our previous results obtained using the *ex vivo* human placental perfusion model suggested that the fetal to maternal transfer of BPAG, even if limited (half that of BPA^35^), might contribute to the removal of BPAG from fetal plasma to a greater extent in humans than in sheep. However, the application of the model to predict BPA and BPAG fetal concentration *vs*. time profiles in rhesus monkeys, considered as a relevant animal model for humans in terms of placental structure^43^, would suggest that our estimated human BPA and BPAG fetal clearances, based on fetal sheep data, are accurate.

Our use of a humanized model to predict plasma concentrations of BPA and BPAG in the late pregnancy human fetus and its mother, under a relevant scheme of BPA dietary exposure for 4 weeks, enabled us to predict major differences between the shapes of the fetal and maternal concentration-time profiles for both BPA and BPAG, in agreement with our previous predictions based on a simplified TK model in fetal sheep^12^. BPA concentrations are predicted to be higher in maternal (14–140 pg/L) than in fetal plasma (11–23 pg/L), due to the efficient fetal to maternal clearance of BPA previously demonstrated in sheep^12^ and humans^35^. The high fetal and maternal clearances of BPA prevented an increase in peak BPA fetal levels over time while the near constant re-entry of BPA, due to BPAG hydrolysis in the fetal compartment, was responsible for the sustained basal BPA concentrations in fetal plasma. The expected maternal and fetal BPA plasma concentrations were of the same order as those predicted (200 and 150 pg/L respectively), for a maternal overall BPA daily intake of 0.5 µg/kg per day by modelling the mother-fetus toxicokinetic interaction^26^. These latter authors suggested that the lower levels of BPA in fetal blood, as compared to maternal, resulted from the ability of the fetus to sulfate BPA and to a limited effect of β-glucuronidase. Our experimental data do not strengthen this hypothesis since they indicate that the enhancement effect of BPAG hydrolysis back to BPA on the systemic exposure of the fetus to BPA is limited by the high fetal clearance of BPA. The predicted values of fetal BPA plasma concentrations are much lower than some reported BPA concentrations in cord serum, these latter ranging from undetectable to 52260 ng/L in the second trimester pregnancy terminations^16^ and 41830 ng/L at full term^44^. As previously discussed, the hypothesis of the immaturity of the UGT system during the second trimester of pregnancy developed by Gerona *et al*.^16^ to explain the high levels of BPA encountered in some samples is not supported by our results.

BPAG concentrations in fetal plasma were predicted to increase over time and attain steady relatively high values of about 29 ng/L after 3 weeks of exposure while the concentrations in the matching maternal plasma exhibited successive peaks and troughs associated with BPA intakes. The expected BPAG plasma concentrations in late pregnancy fetuses were of the same order of magnitude as the BPAG levels in cord serum measured in the above-mentioned studies based on direct assay of BPAG without recourse to a hydrolysis step^16,44^. In these latter two studies, BPAG was detected in 76% and 92% of the umbilical cord serum samples, respectively, with median and maximal concentrations of 120 and 190 ng/L in the second trimester *vs*. 960 and 4850 ng/L at full term. The findings of a recent prospective birth cohort study are also in agreement with our predictions since the mean levels of total BPA in maternal and cord blood samples were 51 ng/L and 46 ng/L^45^. When detected, the unconjugated BPA levels measured in 10 samples of maternal and cord blood, selected among those with the highest concentrations of total BPA, accounted for less than 10% of the total BPA, thereby strengthening the hypothesis that BPA conjugated metabolites represent the dominant form of BPA in both maternal and cord blood.

Our results indicate that, after repeated maternal exposure to a given BPA dose, the BPAG levels in maternal plasma may be highly variable and depend on the time after dosing whereas the BPAG levels in the cord serum are expected to remain relatively steady and reflect the cumulative dose received by the fetus during late pregnancy. Indeed, the slow clearance process of the BPAG trapped in the fetal compartment acts as a damping and delaying mechanism, resulting in less temporal variation. This statement is also supported by the slow decay of BPAG concentrations in fetal plasma when maternal BPA intake is halted. For this reason, the BPAG levels in cord blood could be used as a potential marker of the cumulative BPA dose transferred from the mother to the fetus during late pregnancy. It should be noted that our predictions were based on the average estimated exposure and did not take into account the high interindividual and day-to-day variability in exposure that is reflected by urinary total BPA concentrations in humans^4^. Indeed, we have shown that a single high maternal intake of BPA can have a great and long-lasting impact on both BPAG and basal BPA concentrations in fetal blood whereas any increase in maternal BPA and BPAG concentrations would be expected to be transient. The BPA and BPAG concentrations in cord serum from specific subpopulations can also be expected to be higher than predicted by the current study and will depend on the dose and routes of exposure which may be associated with higher BPA bioavailabilities.

## Conclusions

This study provides critical information about the extent of fetal exposure to BPA and BPAG. Our model simulations predict that the BPAG concentrations in late gestation fetuses remained steady and within the range of values quantifiable by current analytical methods (29 ng/L), based on EFSA’s daily exposure estimates. It should be emphasized that because cord blood concentrations of BPAG are not a matter of controversy due to contamination issues and may not fluctuate as much as plasma concentrations in mothers exposed to episodic BPA intakes, cord blood measurements of BPAG cand be used as a relevant marker of the cumulative dose of BPA received by the fetus during fetal life. Last but not least, the health implications of sustained fetal exposure to BPAG now need to be addressed due to the possible overexposure of some specific fetal tissues resulting from *in situ* hydrolysis of BPAG into BPA.

## Methods

### Animals

All animal procedures were carried out in accordance with accepted standards of humane animal care under agreement number 31-1155545 from the French Ministry of Agriculture. The protocol was approved by the regional ethical committee (Midi-Pyrénées: protocol MP/02/75/11/12). The experiments were carried out on 32 pregnant Lacaune ewes with a mean (±SD) bodyweight of 74 ± 10 kg. One fetus per ewe was chronically catheterized between 105 and 128 days of gestation, as previously described^8^.

### Experimental design

A first experiment was designed to check the dose-proportionality (DP) of BPA and BPAG fetal disposition. DP, that implies that the parameters describing distribution, metabolism and elimination remain constant over a given range of doses, was assessed for BPA doses ranging from about 10 times the highest aggregate environmental exposures in humans i.e. 10 µg/kg.d, to 100 mg/kg.d and for doses ranging from 18 µg/kg.d to 18 mg/kg.d. for BPAG. DP was investigated in 16 female and 14 male fetuses and tested as described by Smith *et al*.^46^ (see Supplementary Material for details of the design of the experiment and data analysis).

In a second experiment in two ewes, designed to refine the sheep feto-maternal model, IV bolus administrations of non-labeled BPA (4.4 µmol/kg, 1 mg/kg) to the mother, and of BPAG-d6 (11.2 µmol, 4.6 mg) to the fetus, were given simultaneously. In this experiment, BPAG was administered in a deuterated form in order to distinguish the BPAG formed from the BPA transferred from the mother to the fetus, from the fetal-administered BPAG and to determine to what extent the first pass conjugation of BPA transferred to the fetus contributed to the fetal exposure to BPAG.

### Test material and treatments

All materials for the preparation of solutions, including materials used for sampling, processing and analysis, were made of glass or BPA-free plastic (polypropylene). BPA (purity 99%), purchased from Sigma-Aldrich (Saint-Quentin Fallavier, France), was extemporaneously dissolved in ethanol/propylene glycol (1/49, vol/vol) at concentrations ranging from 0.00347 to 34.7 mg/mL. BPAG (purity 98%), purchased from Toronto Research Chemicals (Toronto, Canada), was extemporaneously dissolved in ethanol/propylene glycol (1/49, vol/vol) at concentrations ranging from 0.00625 to 31.9 mg/mL. BPAG-d6 (purity 95%), purchased from Toronto Research Chemicals (Toronto, Canada), was extemporaneously dissolved in 0.9% NaCl buffer at 2 mg/mL. Fetal IV administrations were performed via a catheter chronically implanted in the jugular vein. For intravenous infusions, the catheter was connected to a portable syringe pump (Graseby^®^ MS 32), as previously described^47^.

### Sample collection and analysis

Blood samples were collected in heparinized tubes, immediately chilled in ice and centrifuged for 20 min at 3000 g. Plasma was separated and stored at −20 °C until assayed. Analytes were quantified by Acquity ultra performance liquid chromatography coupled to a Xevo triple quadrupole mass spectrometer (Waters, Milford, MA, USA). Chromatographic data were monitored by Targetlynx® software (Water, Milford, MA, USA). BPA and BPAG were simultaneously quantified, without recourse to a hydrolysis step, using a method validated in compliance with FDA guidelines^48^. For BPA-d6 and BPAG-d6 quantification, plasma samples (100 µL) were diluted with 200 µL of acetonitrile and 30 µL of the internal standards BPA-d16 and BPAG13C12 at 10 ng/mL prepared in AcN/H2O (50/50, v/v). The samples were then mixed and centrifuged at 20,000 × g at 4 °C for 10 min, after which the supernatants were transferred to 5 mL glass tubes, evaporated to dryness under a stream of nitrogen and re-suspended in 100 µL of carbonate buffer and 100 µL of a 1 mg/mL solution of dansyl chloride in acetone. After mixing, the solutions were heated for 10 min at 60 °C, the reactions were stopped by cooling the samples on ice and the BPAG-d6-(dansyl)(1) and BPA-d6-(dansyl)(2) derivatives were quantified. The limits of quantification were 0.1 ng/mL (BPA-d6) and 0.25 ng/mL (BPAG-d6). Quality control blanks and quality control samples (at concentrations of 0.25, 2.5 and 75 ng/mL) were used to check the absence of contamination during analysis and the accuracy/precision of the method. Pretreatment samples of maternal blood, fetal blood and amniotic fluid were used as field blanks to assess potential contamination during collection and processing of samples.

### Toxicokinetic analysis

**Figure 7:**
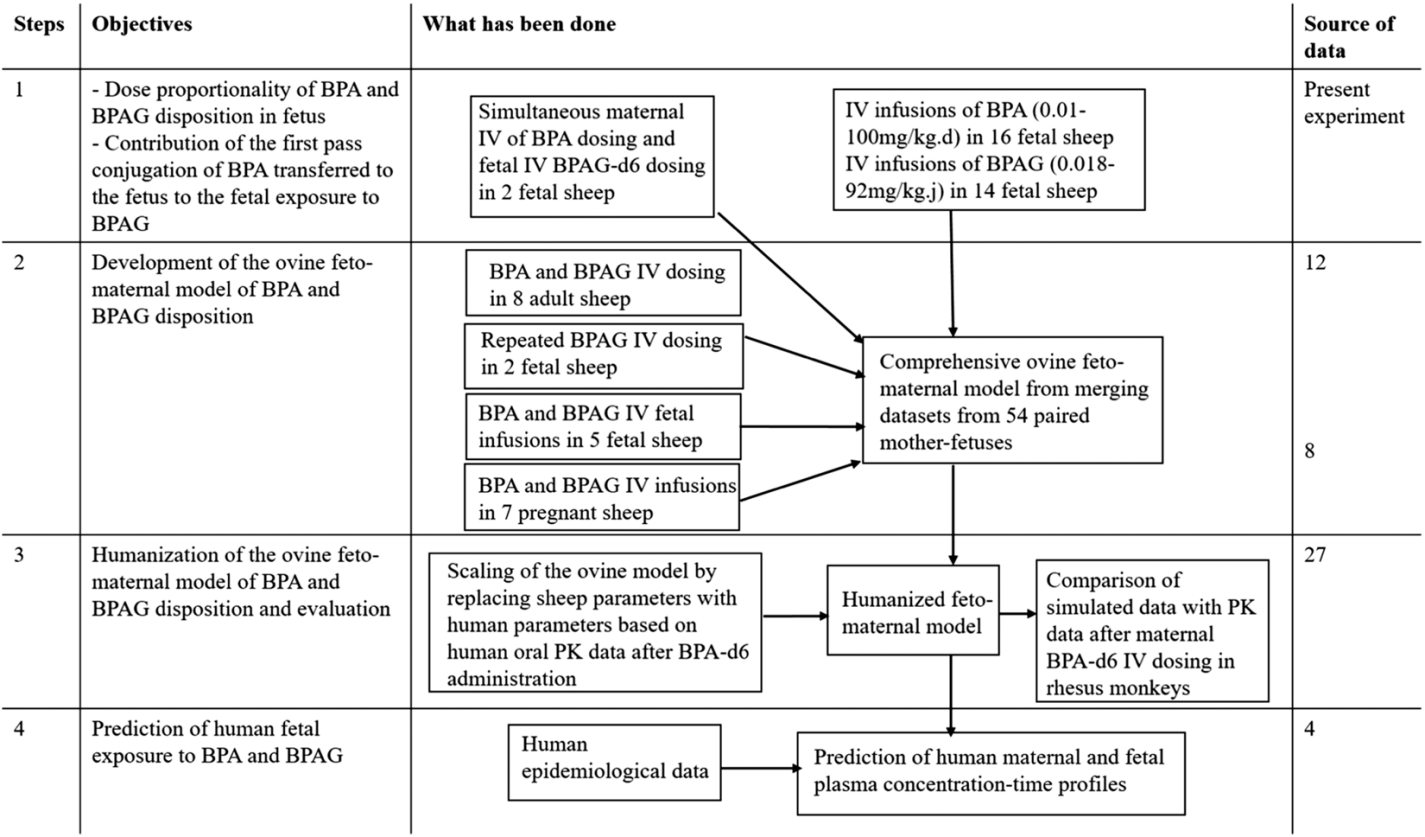
Flowchart of the main steps of the human feto-maternal model development.

The development of a semi-physiological humanized model of BPA and BPAG in the feto-maternal unit was carried out using the Phoenix® NLME (version 6.4; Pharsight, Mountain View, CA, USA) textual model interface with microconstant parameterization. All plasma concentrations were converted into micromolar concentrations before the analysis. All concentrations below the quantification limits were included in the analysis and handled as censored observations according to the M4 method described by Beal^49^. Figure 7 outlines the main steps of the human feto-maternal model development.

### Ovine feto-maternal model development

A previous intravenous BPA and BPAG dosing study with 8 adult sheep was used to characterize the adult (maternal) part of the model^12^. The structure of the adult sheep model and corresponding estimated population parameters (the so-called fixed effects or structural parameters) were then set as the maternal part of the full ovine feto-maternal model. The “full population” ovine feto-maternal model was then developed by combining datasets from the present experiment and from two previous BPA TK studies carried out in pregnant sheep^8,12^ which included a total of 54 paired mother-fetuses. Population modeling is considered an appropriate tool for the meta-analysis of data pooled across different studies^50^. In the study by Gauderat *et al*.^12^, the time courses of BPA and BPAG concentrations in maternal and fetal plasma were measured following IV administration of separate single doses of BPA and BPAG to eight fetal/maternal sheep pairs. In the second part of the study, two fetal sheep received five IV administrations of BPAG, at 6 h intervals. Corbel *et al*.^8^ evaluated the time courses of BPA and BPAG concentrations in maternal and fetal plasma in 5 fetal/maternal sheep pairs following fetal IV infusions of both BPA and BPAG separated by 7 days and in 7 fetal/maternal sheep pairs following maternal IV infusions of both BPA and BPAG, 7 days apart.

### Human feto-maternal model development and evaluation

The maternal part of the ovine feto-maternal model was first re-parameterized with human parameters based on the raw BPA-d6 and total BPA-d6 plasma concentrations obtained after oral BPA-d6 administration in humans via a cookie^27^. BPAG-d6 concentrations in the central compartment were approximated by total BPA-d6 plasma concentrations since the percentage of total BPA-d6 present as BPAG-d6 was largely predominant in human plasma after oral dosing with BPA-d6^27^.

To ensure identifiability of the whole model from this single route of administration, and in the absence of human IV data, the human BPA glucuronidation clearance was fixed at 1.536 L/(kg.h), estimated by allometric approach^30^ and the volume of the human central compartment of BPAG was fixed at the value obtained in sheep (94.5 mL/kg). A humanized feto-maternal model, corresponding to the adult human parameterized model to which had been grafted the full ovine fetal model, was then built by linking the two submodels (adult humanized and ovine fetal models) using the maternal-to-fetal BPA and BPAG clearances estimated in the ovine model. The between-subject variability (BSV) was modeled using an exponential model and the residual unexplained variability (RUV) was modeled using a combined additive and proportional model (see Supplementary Information).

The humanized feto-maternal model was evaluated against published fetal toxicokinetic data from rhesus monkeys as the mechanisms of placental permeability and transfer in rhesus monkeys are analogous to those of humans. The time courses of fetal and maternal plasma concentrations of aglycone BPA-d6 and conjugated BPA-d6 after maternal 100 μg/kg BPA-d6 IV dosing in rhesus monkeys^9^ were compared with the respective values of BPA-d6 and BPAG-d6 plasma concentrations predicted by our model.

### Human BPA and BPAG exposure predictions

The humanized feto-maternal model was employed to simulate the human maternal and fetal BPA and BPAG plasma concentrations resulting from a 4-week exposure scenario relevant to typical environmental exposures. Since diet is considered the main source of human exposure to BPA^4^, the maternal BPA dose was applied to the absorption compartment. The dose selected was the mean daily dose of 36 ng/(kg.d) (11.05 nmol/d for the selected bodyweight of 70 kg) estimated by EFSA for women of childbearing age. This dose was divided into 3 sub-doses delivered at the respective intervals of 5, 7 and 12 h to mimic three meals per day. A single dose corresponding to the 95th percentile estimation of 182 ng/(kg.d) (mean-95th percentile, 55.85 nmol) was applied once after three weeks of daily exposure to the mean dose to assess the impact of occasional exposure to a high dose on the time courses of BPA and BPAG concentrations in maternal and fetal plasma.

The datasets generated during and/or analysed during the current study are available from the corresponding author on reasonable request.

## Electronic supplementary material

Supplementary information
