# Supplementary material for: Prediction of human prenatal exposure to bisphenol A and bisphenol A glucuronide from an ovine semi-physiological toxicokinetic model
Source: Sci Rep. 2017 Nov 10;7:15330. doi: 10.1038/s41598-017-15646-5 (PMC5681680; doi:10.1038/s41598-017-15646-5)
Supplement: Supplementary file 1 — Supplementary information [file 41598_2017_15646_MOESM1_ESM.pdf]

SUPPLEMENTARY INFORMATION

Prediction of human prenatal exposure to bisphenol A and bisphenol A glucuronide from an ovine semi-physiological toxicokinetic model

Glenn Gauderat[ab], Nicole Picard-Hagen[a], Pierre-Louis Toutain[a], Servien Rémi[a], Catherine Viguie[a], Sylvie Puel[a], Marlène Z. Lacroix[a], Tanguy Corbel[a], Alain Bousquet-Melou[a] and Véronique Gayrard[a]

[a]Toxalim, Université de Toulouse, INRA (Institut National de la Recherche Agronomique), INP (Institut National Polytechnique de Toulouse) –ENVT (Ecole Nationale Vétérinaire de Toulouse), Toulouse, France
[b]Agreenium's International Research School (EIR-A), Paris, France

Corresponding author : Véronique Gayrard, UMR1331 Toxalim, Ecole Nationale Vétérinaire de Toulouse, Laboratoire de Physiologie, 23 chemin des Capelles, BP 87614, 31076 Toulouse cedex 3, France, Tel. : 33 (0)5 61 19 39 18, Fax. : 33 (0)5 61 19 39 17, E-mail : v.gayrard@envt.fr

Contents **Page**

Supplementary Figure S1. Test of linearity of the disposition of BPA and BPAG...................................... 3

Supplementary Figure S2. Test of linearity of the disposition of BPA and BPAG...................................... 4

Supplementary Figure S3. Semilogarithmic plots of mean (lines) and individual (dots) concentration-time profiles (µM) of BPA, BPAG, BPA-d6 and BPAG-d6 in maternal and fetal plasma following simultaneous IV administrations of BPAG-d6 to fetus (11.2µmol, 4.6 mg) and BPA to the mother (4.4 µmol/kg, 1 mg/kg)... 5

Supplementary Figure S4. Compartmental representation of the model developed in adult sheep. 6

Supplementary Figure S5. Compartmental representation of the feto-maternal sheep model. 7

Supplementary Figure S6. Compartmental representation of the adult human model................................. 8

Supplementary Table S1. Parameter estimates and associated confidence intervals obtained when fitting fetal plasma concentrations vs. fetal infusion rates to a simple linear model... 9

Supplementary Table S2. BPA and BPAG toxicokinetic parameter estimates obtained for adult sheep. .. 10

Supplementary Table S3. BPA and BPAG toxicokinetic parameter estimates obtained for the feto-maternal sheep unit... 11

Supplementary Table S4. BPA and BPAG toxicokinetic parameter estimates obtained for adult humans. 13

Supplementary Method: Dose linearity analysis 14

Supplementary Method: Between subject and residual variability modeling 15

Supplementary References 15

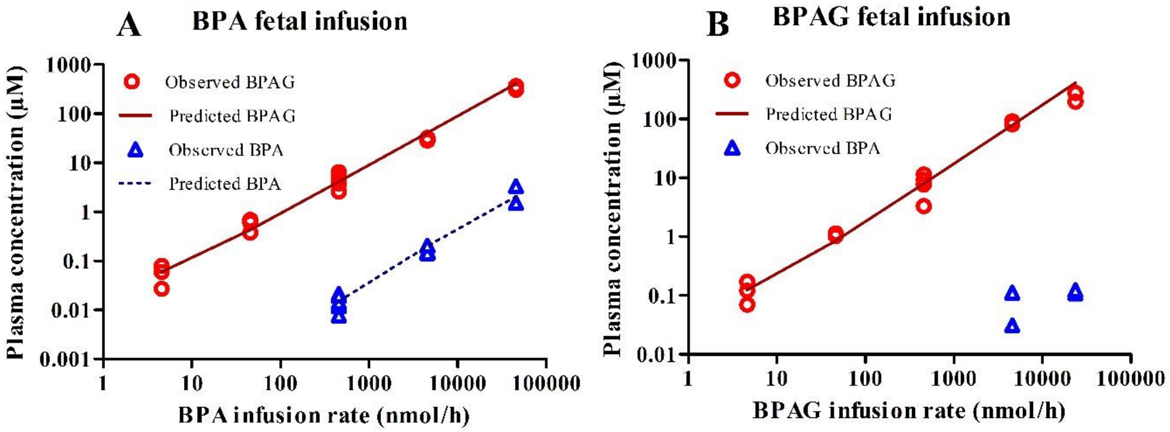

Supplementary Figure S1. Test of linearity of the disposition of BPA and BPAG.
Linear model-predicted and observed fetal BPAG and BPA plasma concentrations three days after the beginning of a fetal IV infusion of non-labeled BPA at doses of 4.6 nmol/h (0.01 mg/kg.d, n=3), 45.7 nmol/h (0.1 mg/kg.d, n=3), 456.6 nmol/h (1 mg/kg.d, n=5), 4566.4 nmol/h (10 mg/kg.d, n=3) or 45664.1 nmol/h (100 mg/kg.d, n=3) (A) and three days after the beginning of a fetal IV infusion of non-labeled BPAG at doses of 4.6 nmol/h (0.018 mg/kg.d, n=3), 46.4 nmol/h (0.18 mg/kg.d, n=3), 456.2 nmol/h (1.8 mg/kg.d, n=4), 4562.1 nmol/h (18 mg/kg.d, n=2) or 23712 nmol/h (92 mg/kg.d, n=2) (B). The nominal fetal body weight was arbitrarily fixed at 2.5 kg[1]. Two to four serial fetal blood samples (1 mL) were drawn before beginning the infusions and 3 days after beginning the infusions to determine BPA and BPAG plasma concentrations.

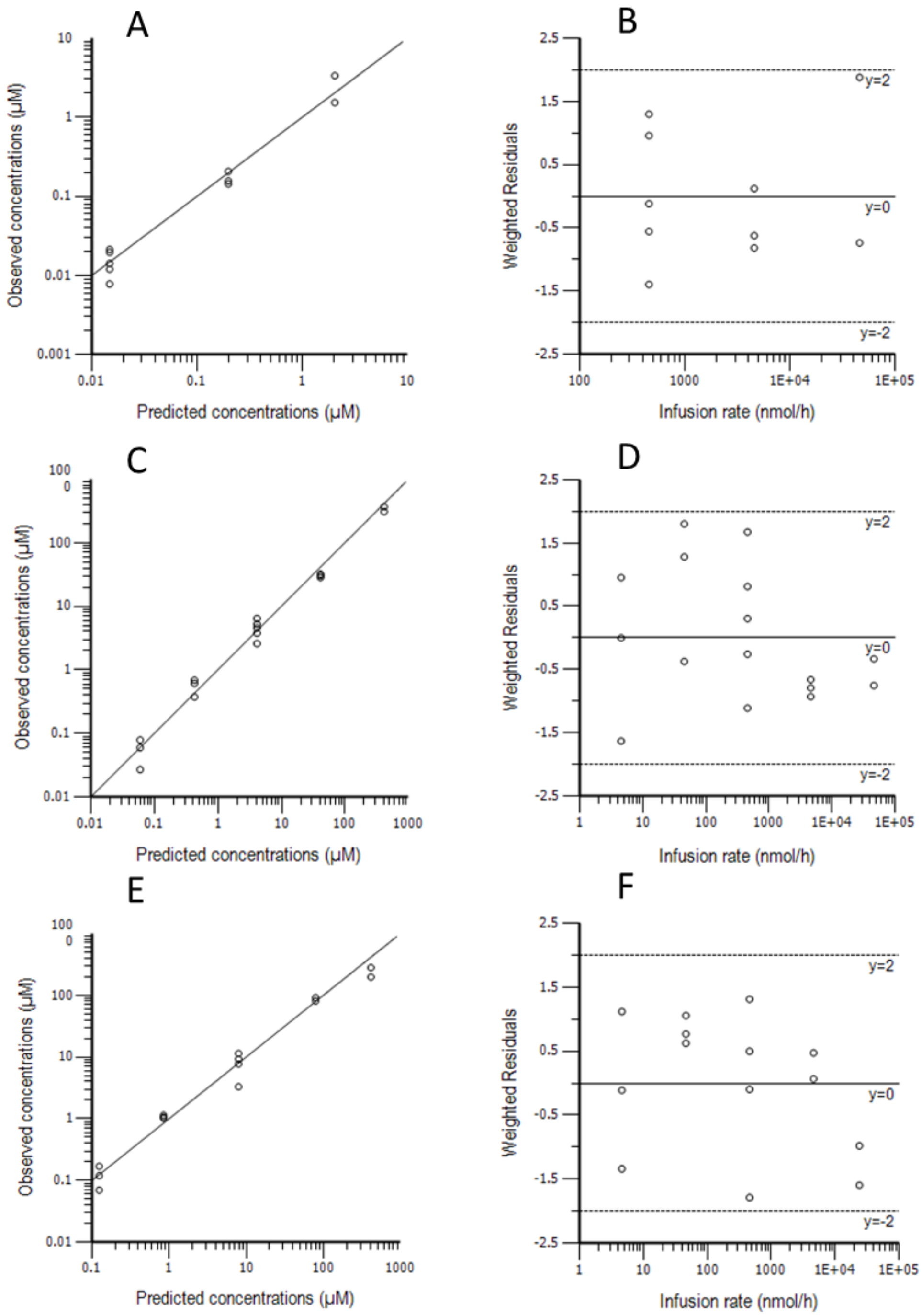

Supplementary Figure S2. Test of linearity of the disposition of BPA and BPAG.
Diagnostic plots obtained by fitting fetal plasma concentrations vs infusion rates to a simple linear model. Observed *vs*. predicted fetal plasma concentrations (left panels) and associated weighted residual *vs*. infusion rate plots (right panels) are presented for BPA infusion *vs*. BPA fetal plasma concentrations (A and B), BPA infusion *vs*. BPAG fetal plasma concentrations (C and D) and BPAG infusion *vs*. BPAG fetal plasma concentrations (E and F).

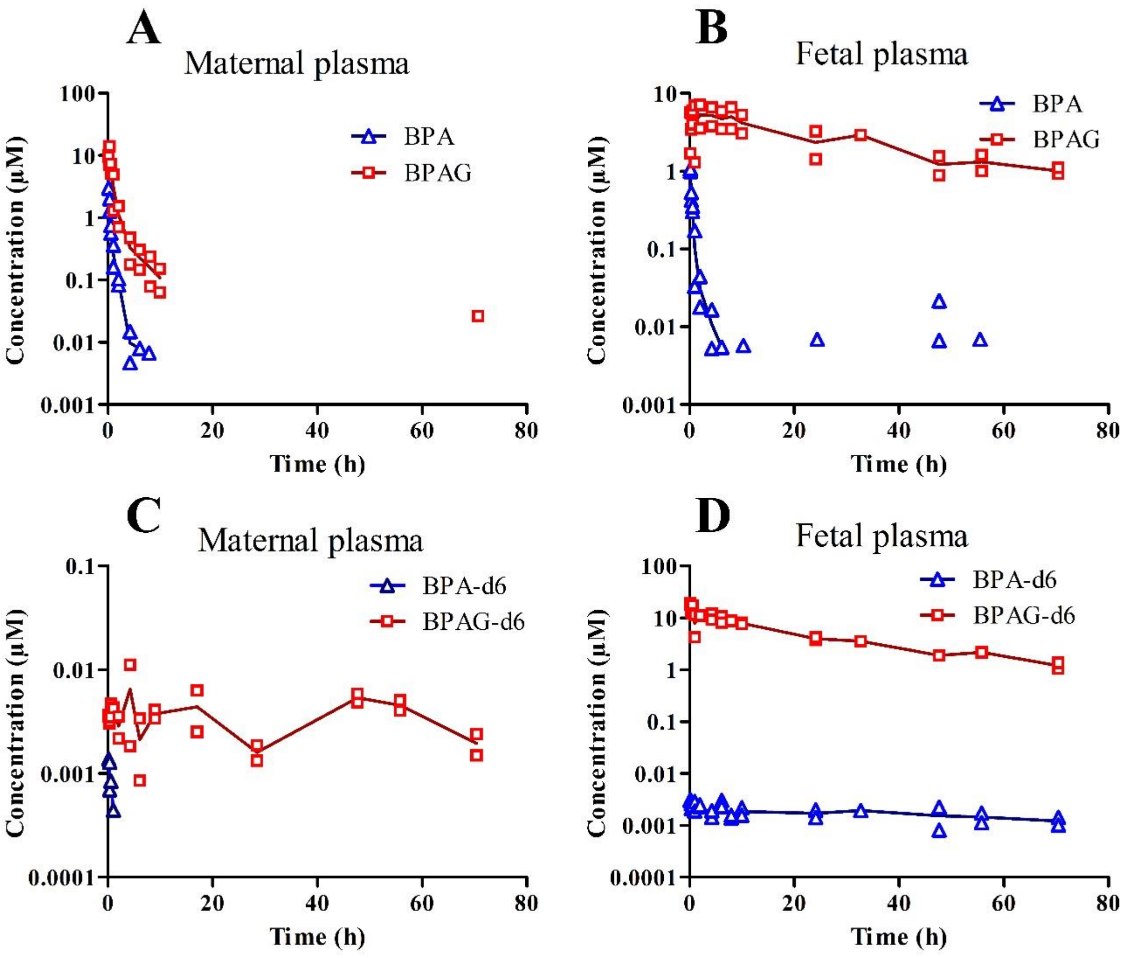

Supplementary Figure S3. Semilogarithmic plots of mean (lines) and individual (dots) concentration-time profiles (µM) of BPA, BPAG, BPA-d6 and BPAG-d6 in maternal and fetal plasma following simultaneous IV administrations of BPAG-d6 to fetus (11.2µmol, 4.6 mg) and BPA to the mother (4.4 µmol/kg, 1 mg/kg).

Fetal and maternal blood samples (0.5mL) were taken before and at 7, 15, 30, 60, and 120 min, every 2 h for 8 h and at 24, 34, 48, 56 and 70 h after the administrations. Non-labeled BPA and BPAG plasma concentrations resulting from the maternal BPA administration are presented in the top panels for the mother (A) and the fetus (B). BPA-d6 and BPAG-d6 plasma concentrations resulting from the fetal BPAG-d6 administration are presented in the bottom panels (C and D).

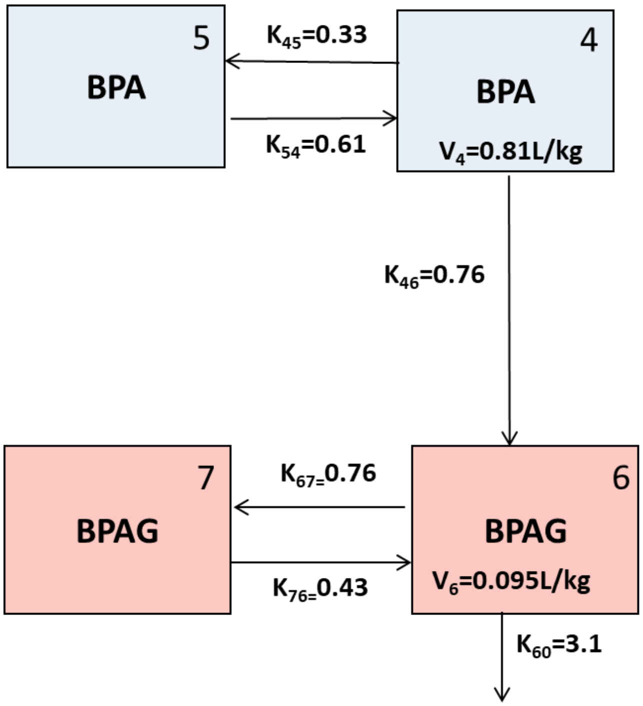

Supplementary Figure S4. Compartmental representation of the model developed in adult sheep. The model includes both a central and a peripheral compartment respectively designated 4 and 5 for BPA and as 6 and 7 for BPAG with a glucuronoconjugation rate constant between BPA and BPAG central compartments designated K46. The central and peripheral BPA and BPAG compartments are interconnected with rate constants of transfer, respectively represented by K45 and K54 for BPA and as K67 and K76 for BPAG. The BPAG elimination constant rate is represented by K60. Values above the arrows represent the transfer rates between compartments (per hour). V4 and V6 represent the respective volumes of the central compartments for BPA and BPAG.

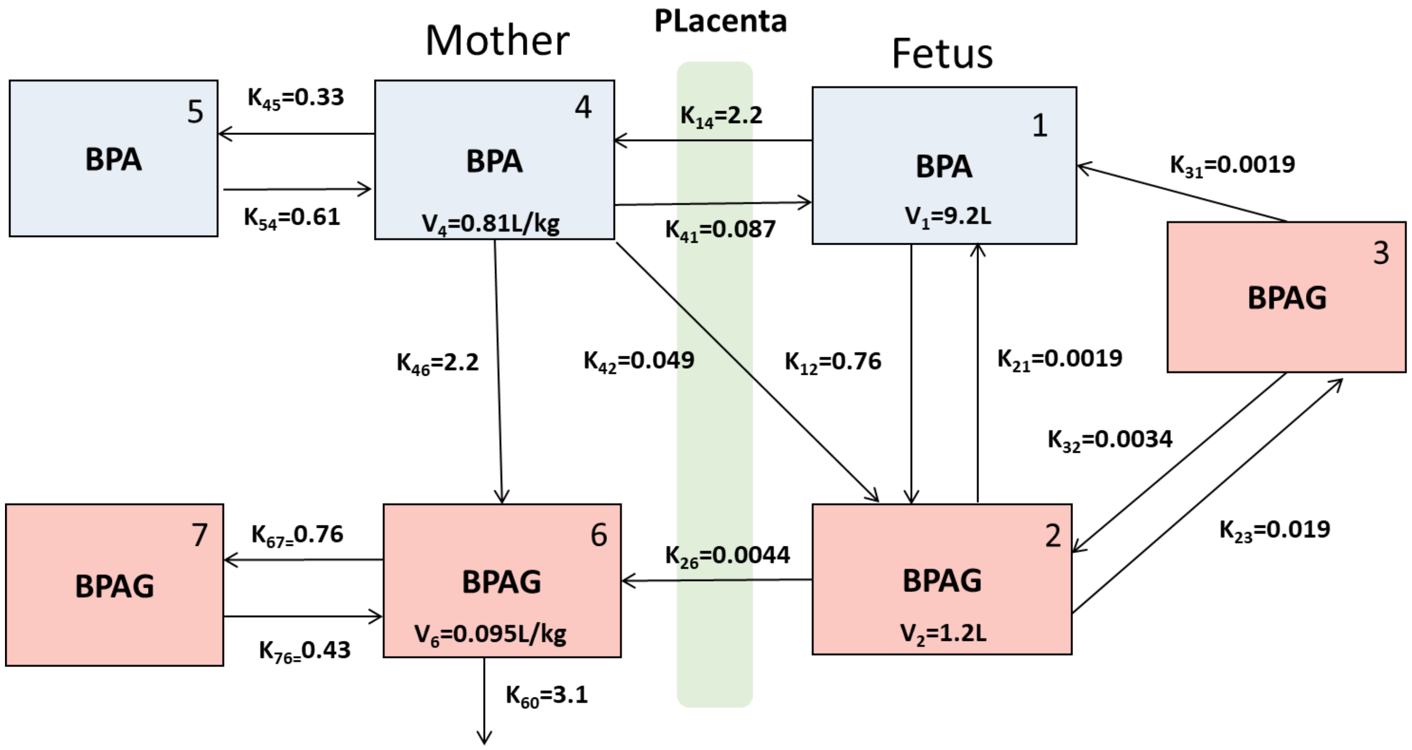

Supplementary Figure S5. Compartmental representation of the feto-maternal sheep model.
The fetal part of the model includes a central compartment for both BPA and BPAG represented by 1 and 2 and a peripheral compartment for BPAG represented by 3. The maternal part of the model includes both a central and a peripheral compartment indicated respectively as 4 and 5 for BPA and as 6 and 7 for BPAG. Values above the arrows represent rates of transfer between compartments (per hour). V1 and V4 represent the respective volumes of the fetal and maternal central compartments for BPA. V2 and V6 represent the respective volumes of the fetal and maternal central compartments for BPAG.

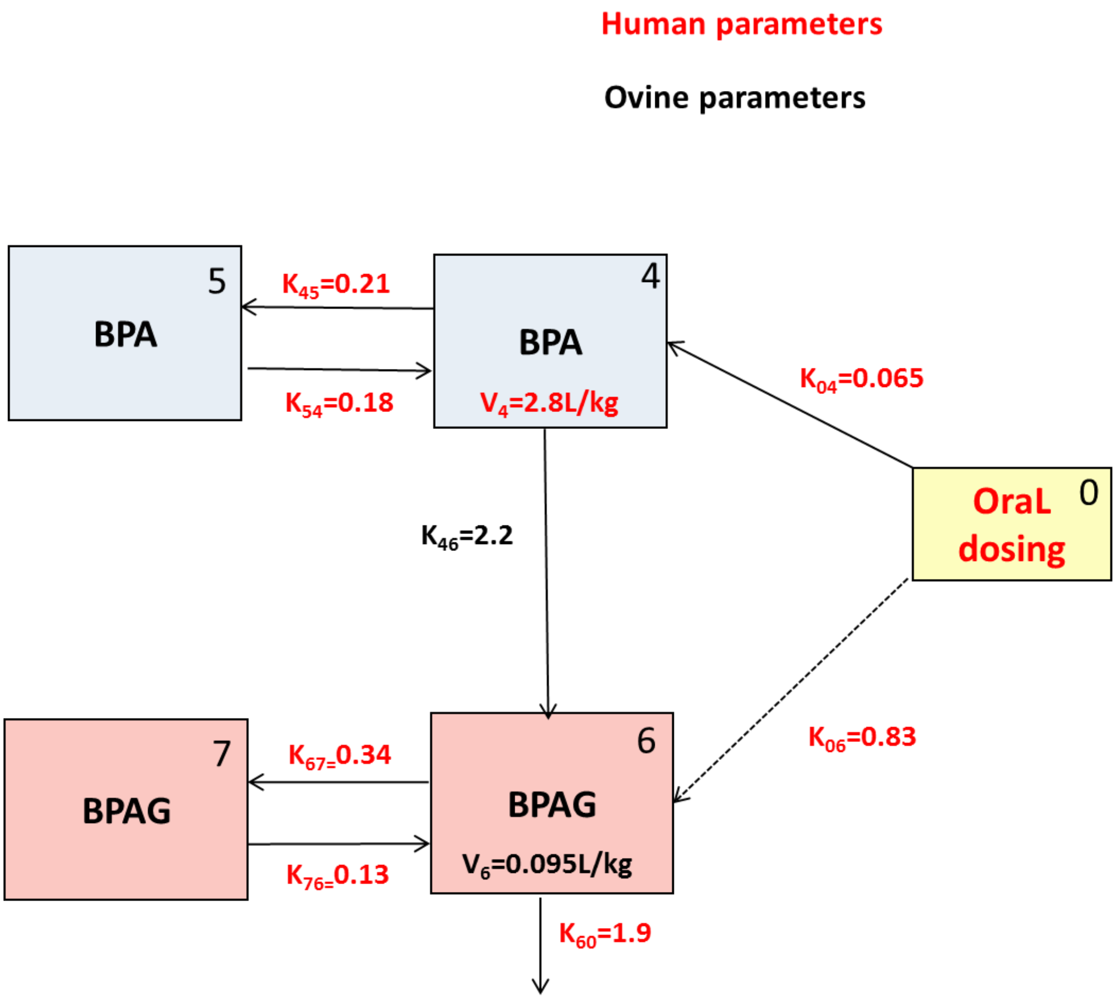

Supplementary Figure S6. Compartmental representation of the adult human model.
The model structure consisted of the adult part of the ovine model to which was added an absorption sub-model. The oral BPA dose is administered in the absorption compartment, designated 0, and linked to the BPA and the BPAG central compartments with rate constants of transfer to describe respectively BPA bioavailability (K04) and BPA first-pass conjugation (K06). Red parameters were estimated from human data while black parameters were fixed at the values estimated in sheep.

Supplementary Table S1. Parameter estimates and associated confidence intervals obtained when fitting fetal plasma concentrations vs. fetal infusion rates to a simple linear model. α and β1 represent the intercept and slope of the linear model. For α, the confidence interval included 0 indicating that α is not significantly different from 0.

Adjusted data	Parameter	Estimate	Coefficient of variation (%)	2.5% confidence interval	97.5% confidence interval
**BPA fetal plasma concentrations vs BPA fetal infusion rate**	α	-5.5E-03	-6.4E+01	-1.4E-02	2.9E-03
	β1	4.5E-05	1.5E+01	2.9E-05	6.0E-05
	SD	3.4E-01	2.5E+01	1.4E-01	5.4E-01
**BPAG fetal plasma concentrations vs BPA fetal infusion rate**	α	1.9E-02	6.7E+01	-8.6E-03	4.6E-02
	β1	9.0E-03	9.7E+00	7.1E-03	1.1E-02
	SD	3.4E-01	2.0E+01	1.9E-01	4.8E-01
**BPAG fetal plasma concentrations vs BPAG fetal infusion rate**	α	4.4E-02	5.6E+01	-1.0E-02	9.9E-02
	β1	1.7E-02	1.0E+01	1.3E-02	2.1E-02
	SD	3.3E-01	2.1E+01	1.8E-01	4.8E-01

Supplementary Table S2. BPA and BPAG toxicokinetic parameter estimates obtained for adult sheep.

NA: Not Applicable (no random component included in the model for the parameters)

**Parameter**		**Population estimate**	**Units**	**Coefficient of variation for between subject variability (%)**	**Eta shrinkage**
**V4**		0.8148	L/kg	9.9	0.32
**V6**		0.0945	L/kg	16.6	0.14
**K60**		3.1394	L/h	21.8	0.04
**K67**		0.7558	1/h	NA	NA
**K76**		0.4253	1/h	NA	NA
**K46**		2.1533	1/h	14.8	0.08
**K45**		0.3279	1/h	32.7	0.23
**K54**		0.6051	1/h	15.8	0.18
**BPAMultStdev**		19.84	CV (%)		
**BPAGMultStdev**		20.61	CV (%)		
**stdevBPA**		0.0017	µM		
**stdevBPAG**		0.1122	µM		

V4: volume of the maternal (or adult) BPA central compartment
V6: volume of the maternal (or adult) BPAG central compartment
K60: maternal BPAG elimination rate
K67: maternal BPAG output distribution rate
K76: maternal BPAG input distribution rate
K46: maternal BPA conjugation rate
K45: maternal BPA output distribution rate
K54: maternal BPA input distribution rate
BPAMultStdev: standard deviation of the multiplicative error term ε(1) for BPA concentrations (expressed as a coefficient of variation)
BPAGMultStdev: standard deviation of the multiplicative error term ε(1) for BPAG concentrations (expressed as a coefficient of variation)
stdevBPA: standard deviation of the additive error term ε(2) for BPA concentrations (same unit as BPA concentrations)
stdevBPAG: standard deviation of the additive error term ε(2) for BPAG concentrations (same unit as BPAG concentrations)

Supplementary Table S3. BPA and BPAG toxicokinetic parameter estimates obtained for the feto-maternal sheep unit.
NA: Not Applicable

Parameter	Population estimate	Units	Coefficient of variation for between subject variability (%)	Eta shrinkage
**K21**	0.0019	1/h	NA	NA
**V1**	0.9153	L	81.6	0.348
**V2**	1.166	L	53.2	0.170
**K14**	2.2218	1/h	NA	NA
**K12**	0.7637	1/h	NA	NA
**K41**	0.0868	1/h	NA	NA
**K32**	0.0034	1/h	NA	NA
**K23**	0.0190	1/h	76.4	0.324
**K42**	0.0491	1/h	NA	NA
**K31**	0.0019	1/h	NA	NA
**K26**	0.0044	1/h	NA	NA
**V4 (frozen)**	814.8	mL/kg	NA	NA
**V6 (frozen)**	94.5	mL/kg	NA	NA
**K60 (frozen)**	3.14	1/h	67.0	0.234
**K45 (frozen)**	0.76	1/h	NA	NA
**K54 (frozen)**	0.43	1/h	NA	NA
**K46 (frozen)**	2.15	1/h	68.0	0.286
**K45 (frozen)**	0.33	1/h	NA	NA
**K54 (frozen)**	0.61	1/h	NA	NA
**BPAMultStdev**	40.04	CV (%)		
**BPAGMultStdev**	33.15	CV (%)		
**BPA-d6MultStdev**	0.85	CV (%)		
**BPAG-d6MultStdev**	46.31	CV (%)		
**stdevBPA**	0.0040	µM		
**stdevBPAG**	0.0196	µM		
**stdevBPA-d6**	0.0011	µM		
**stdevBPAG-d6**	0.0025	µM		

The population estimates of parameters indicated as "(frozen)" were incorporated as previously-estimated fixed values.

K21: fetal BPAG hydrolysis rate from the central compartment
K31: fetal BPAG hydrolysis rate from the peripheral compartment
V1: volume of the fetal BPA central compartment

V2: volume of the fetal BPAG central compartment
K41: fetal-to-maternal BPA transfer rate
K12: fetal BPA conjugation rate
K41: maternal-to-fetal BPA transfer rate
K32: fetal BPAG input distribution rate
K23: fetal BPAG output distribution rate
K42: maternal-to-fetal BPA conjugation rate
K26: fetal-to-maternal BPAG transfer rate
V4: volume of the maternal (or adult) BPA central compartment
V6: volume of the maternal (or adult) BPAG central compartment
K60: maternal BPAG elimination rate
K67: maternal BPAG output distribution rate
K76: maternal BPAG input distribution rate
K46: maternal BPA conjugation rate
K45: maternal BPA output distribution rate
K54: maternal BPA input distribution rate
BPAMultStdev: standard deviation of the multiplicative error term ε(1) for BPA concentrations (expressed as a coefficient of variation)
BPAGMultStdev: standard deviation of the multiplicative error term ε(1) for BPAG concentrations (expressed as a coefficient of variation)
BPA-d6MultStdev: standard deviation of the multiplicative error term ε(1) for BPA-d6 concentrations, (expressed as a coefficient of variation)
BPAG-d6MultStdev: standard deviation of the multiplicative error term ε(1) for BPAG-d6 concentrations (expressed as a coefficient of variation)
stdevBPA: standard deviation of the additive error term ε(2) for BPA concentrations (same unit as BPA concentrations)
stdevBPAG: standard deviation of the additive error term ε(2) for BPAG concentrations (same unit as BPAG concentrations)
stdevBPA-d6: standard deviation of the additive error term ε(2) for BPA-d6 concentrations (same unit as BPA-d6 concentrations)
stdevBPAG-d6: standard deviation of the additive error term ε(2) for BPAG-6 concentrations (same unit as BPAG-d6 concentrations)

Supplementary Table S4. BPA and BPAG toxicokinetic parameter estimates obtained for adult humans.

Parameter	Population estimate	Units	Coefficient of variation for between subject variability (%)	Eta shrinkage
**K42**	0.826	1/h	7.85	0.537
**K04**	0.065	1/h	33.1	0.0399
**V4**	2.803	L/kg	40.6	0.0589
**V6 (frozen)***	0.095	L/kg	NA	NA
**K60**	1.903	1/h	6.7	0.471
**K67**	0.337	1/h	23.6	0.415
**K76**	0.129	1/h	20.5	0.538
**K45**	0.208	1/h	72.8	0.0910
**K54**	0.184	1/h	5.10	0.752
**K46 ****	0.548	1/h	NA	NA
**BPA-d6MultStdev**	35.7	CV (%)		
**BPAG-d6MultStdev**	42.3	CV (%)		
**stdevBPA-d6**	0.001	µM		
**stdevBPAG-d6**	0.059	µM		

Analyzed raw data were from Thayer et al.[2]. *The population estimate of V6 was fixed at the corresponding ovine estimation. **The parameter K46 was defined a priori as the ratio of the 1.536 L/kg.h clearance[30] divided by the BPA central volume (V4).

K42: maternal-to-fetal BPA conjugation rate
V4: volume of the maternal (or adult) BPA central compartment
V6: volume of the maternal (or adult) BPAG central compartment
K60: maternal BPAG elimination rate
K67: maternal BPAG output distribution rate
K76: maternal BPAG input distribution rate
K46: maternal BPA conjugation rate
K45: maternal BPA output distribution rate
K54: maternal BPA input distribution rate
BPA-d6MultStdev: standard deviation of the multiplicative error term ε(1) for BPA-d6 concentrations (expressed as a coefficient of variation)
BPAG-d6MultStdev: standard deviation of the multiplicative error term ε(1) for BPAG-d6 concentrations (expressed as a coefficient of variation)
stdevBPA-d6: standard deviation of the additive error term ε(2) for BPA-d6 concentrations (same unit as BPA-d6 concentrations)
stdevBPAG-d6: standard deviation of the additive error term ε(2) for BPAG-6 concentrations (same unit as BPAG-d6 concentrations)

Supplementary Method: Dose linearity analysis

Fetal BPA and BPAG plasma concentrations at the end of the infusion were fitted against the BPA or BPAG infusion rates using a linear model of the form:

Equation (1): $Css = \alpha + \beta_1(\tau)$

Where Css was the mean fetal plasma concentration (in µM) at the end of the infusion, τ was the rate of intravenous fetal infusion (in nmol/h) and β_1 the linear component of the model.

The model was resolved using a proportional residual error model of the form:

Equation (2): $CssObs = Css \times (1 + \varepsilon)$

Where Css and $CssObs$ were the predicted and observed fetal plasma concentrations and ε was the residual error.

Dose-proportionality was accepted provided that the 95 % confidence interval of the estimated term α included 0 when fitting the data to the simple linear model[3].

Supplementary Method: Between subject and residual variability modeling

The between subject variability (BSV) and the residual unexplained variability (RUV) were modeled in the same way for all the models.

The BSV was modeled using an exponential model of the form:

Equation (3): $\theta_i = \theta_1 \times Exp(\eta_i)$

With θ_i (designated theta) the value of TK parameter in the ith animal, θ_1 the typical population value of this parameter and η_i the deviation (designated eta) associated with the ith animal from the corresponding population value.

It was assumed that etas were not correlated and no covariance terms were considered.

The RUV was modeled using combined additive and proportional errors of the form:

Equation (4): $Cobs = Cpred + \varepsilon(1) + Cpred \times \varepsilon(2)$

With $Cobs$ and $Cpred$ the observed and predicted concentrations and $\varepsilon(1)$ and $\varepsilon(2)$ the additive and multiplicative residual terms.

One residual error model per analyte was defined (BPA, BPAG, BPA-d6 and BPAG-d6).

Supplementary References

1. Bazer, F. W., Spencer, T. E. & Thatcher, W. W. Growth and development of the ovine conceptus. *J. Anim. Sci.* **90,** 159–170 (2012).
2. Thayer, K. A. *et al.* Pharmacokinetics of bisphenol A in humans following a single oral administration. *Environ. Int.* **83,** 107–115 (2015).
3. Smith, B. P. *et al.* Confidence interval criteria for assessment of dose proportionality. *Pharm. Res.* **17,** 1278–1283 (2000).
